# Infection-associated Hemophagocytic Lymphohistiocytosis: An Unusual Clinical Masquerader

**DOI:** 10.7759/cureus.2472

**Published:** 2018-04-12

**Authors:** Awais Abbas, Mohammad Raza, Aamina Majid, Yumna Khalid, Syed Hamza Bin Waqar

**Affiliations:** 1 Department of Pediatrics, Civil Hospital Karachi, Dow University of Health Sciences (DUHS), Karachi, PAK; 2 Pediatric Department, Civil Hospital Karachi, Dow University of Health Sciences (DUHS), Karachi, PAK; 3 Medical Student, Civil Hospital Karachi, Dow University of Health Sciences (DUHS), Karachi, PAK

**Keywords:** hemophagocytic lymphohistiocytosis, cytopenia, salmonella typhi, typhoid fever, hyperferritinemia

## Abstract

Hemophagocytic lymphohistiocytosis (HLH) secondary to an infection is a great impersonator. It is caused by hyperimmune activation, which leads to a wide array of hematological abnormalities. If the disease is untreated, it is usually fatal. We report the case of a four-year-old girl who presented to our tertiary care hospital with high-grade fever, frequent loose stools, and bleeding from the lips and gums. Investigations showed pancytopenia, hyperferritinemia, hypofibrinogenemia, and hypertriglyceridemia whereas the bone marrow biopsy revealed hemophagocytosis with trilineage suppression. Blood cultures grew Salmonella typhi. After ruling out other possibilities, the diagnosis of HLH was made as per the HLH-2004 diagnostic criteria. The patient responded well to culture-sensitive antibiotics and supportive treatment. We discuss the diagnosis and clinical course of this unique case and strive to create awareness about secondary HLH induced by common diseases, such as typhoid fever.

## Introduction

Typhoid fever is a systemic illness most commonly caused by the bacterium Salmonella enterica serotype typhi. It has the highest occurrence and morbidity in Asia and constitutes a major disease burden [1]. It commonly presents with fever, malaise, headache, anorexia, abdominal pain, and diarrhea. Far less common, however, is the occurrence of Hemophagocytic lymphohistiocytosis (HLH) secondary to typhoid fever.

HLH is a rare hyperinflammatory condition characterized by hematological and immunological changes with hemophagocytosis and significant functional alterations in natural killer cells. While the primary form of the disease is familial, the secondary form can occur secondary to an infection, malignancy, autoimmune disease, or immune deficiency state. This life-threatening condition is uniformly fatal if left untreated. Several infectious agents have been implicated in causing HLH but typhoid fever has been documented only in a handful of cases.

Here, we present the case of a pediatric patient who presented with fever, diarrhea, and bleeding from the gums and tested positive for Salmonella typhi. Her bone marrow biopsy result led us to the diagnosis of HLH. Timely diagnosis and appropriate treatment saved this patient’s life from a deadly but potentially reversible disease.

## Case presentation

A previously healthy four-year-old girl was brought to the emergency department of Civil Hospital, Karachi, with fever and diarrhea for two weeks and bleeding from the gums and lips for one day. The fever was sudden in onset, high grade, and associated with chills and rigors. Additionally, the diarrhea was non-bloody and watery, occurring four to five times a day. The patient also suffered from generalized body aches, nausea, and decreased appetite. The patient denied abdominal pain, vomiting, constipation, black tarry stools, tea-colored urine, ear discharge, or a cough. There was no history of recent vaccination or travel, and her immunization record was up to date. Birth and past history were unremarkable, as was the family history.

On admission, she was conscious but irritable with the following vitals: A temperature of 38.9⁰C, blood pressure of 90/60 mmHg, heart rate of 128 beats per minute (BPM), respiratory rate of 26/min, and oxygen saturation of 98%. The capillary refill time (CRT) was <2 sec. The physical examination was notable for pallor and dry, cracked lips. Bruises were found on the limbs, and there was no history of trauma or child abuse. There were no signs of jaundice, edema, visceromegaly, lymphadenopathy, or skin rashes. Abdominal, respiratory, cardiovascular, and central nervous systems were normal on physical examination.

The full blood count revealed pancytopenia with a hemoglobin of 7.5 mg/dl, a white blood cell (WBC) count of 1.7×109/L, and a platelet count of 55×109/L. The differentiated leukocyte count by automated cell counter showed 56% neutrophils, 41% lymphocytes, and 3% monocytes. The reticulocyte count was 0.4%. A prothrombin time (PT) of 10 seconds and an activated partial thromboplastin time (APTT) of 19 seconds were recorded. Renal function tests were normal but liver function tests were deranged (Table 1). Viral serology for hepatitis viruses B and C, human immunodeficiency virus (HIV), dengue virus non-structural protein 1 (NS1), and dengue IgM were all negative. There were no malarial parasites on peripheral blood smear. The laboratory test results carried out at our hospital are shown in Table 1.

**Table 1:** The laboratory tests during the hospital stay TLC: Total leukocyte count, PT: Prothrombin time, APTT: Activated partial thromboplastin time, CRP: C-reactive protein, BUN: Blood urea nitrogen, ALT: Alanine aminotransferase, AST: Aspartate aminotransferase, LDH: Lactate dehydrogenase

INVESTIGATIONS	RESULTS	REFERENCE RANGE
Hemoglobin	7.5 g/dl	11-13g/dl (children)
Platelet count	55 x 10^9^/L	150–400 × 10^9^/L
TLC	1.7x 10^9^/L	4-11 x 10^9^/L
Differentiated leukocyte count	N56L41M3E0	
Reticulocytes	0.4%	0.5-2%
PT	10 sec	11-14 sec
APTT	19 sec	26-40 sec
CRP	39.5 mg/L	<10 mg/L
Serum creatinine	0.4 mg/dl	0.5-1.2 mg/dl
BUN	15 md/dl	7-20 mg/dl
Serum sodium	142 mmol/L	135-145 mmol/L
Serum potassium	4.1 mmol/L	3.5-5.5 mmol/L
Plasma albumin	4.7 g/dl	3.5-5.5 g/dl
Serum total bilirubin	0.23 mg/dl	0.3-1.3 mg/dl
ALT	125 U/L	5-40 U/L
AST	137 U/L	5-40 U/L
Serum alkaline phosphatase	193 U/L	35-110 U/L
Serum LDH	1383 U/L	140-280 U/L
Fasting plasma triglycerides	343 mg/dl	30-200 mg/dl
Fibrinogen	0.9 g/L	2-4 g/L
Serum ferritin	1935 ng/ml	29-248 ng/ml

The patient was resuscitated with intravenous (IV) fluids. Empirical antibiotic therapy was started with intravenous ceftriaxone. The patient remained febrile during the initial days of therapy and her diarrhea did not improve. Additionally, her blood cell lineages continued to decline (Table 2). The blood culture showed the growth of Salmonella enterica serotype typhi. The disk diffusion method for susceptibility disclosed the Salmonella typhi to be susceptible to ceftriaxone, ciprofloxacin, vancomycin, tazobactam, and norfloxacin but resistant to nalidixic acid and amoxicillin. The patient’s cell lines responded to IV ceftriaxone; however, the febrile episodes persisted along with loose stools. So, we switched the regimen to IV tazobactam and vancomycin with supplementation of folic acid, vitamin B12, and zinc.

**Table 2:** Declining hematological parameters at the beginning of the treatment TLC: Total leukocyte count

	Day 1	Day 4	Day 8	Day 12
Hemoglobin (g/dL)	7.5	7.3	7	6.7
TLC (×10^9^/L)	1.7	1.7	1.6	1.6
Neutrophils (%)	56	50	35	28
Lymphocytes (%)	41	48	60	64
Monocytes (%)	3	2	5	8
Platelets (×10^9^/L)	55	50	43	34

Bone marrow aspiration and trephine biopsy were performed to explore pancytopenia. We then performed a bone marrow cytology, which showed suppressed trilineage hematopoiesis. Erythroid and myeloid precursors were seen scattered throughout the smear, and lymphocytes were prominent. An increased hemophagocytic activity was also noted and blast cells were found to be <5%. This picture was suggestive of HLH.

Further pertinent investigations were carried out for confirmation. Serum ferritin and triglycerides were quantified and values were found to be 1935 ng/ml and 343 mg/dl, respectively. Fibrinogen levels were low with a value of 0.9 g/L. These findings, together with the clinical picture and laboratory data, were compatible with a diagnosis of secondary HLH. Primary HLH was considered unlikely and there was no family history of HLH. The patient responded to antibiotics after 14 days. During this time, she was also given supportive management with regular platelet and packed cell transfusions. After this therapy, the pancytopenia improved, as shown in (Table 3).

**Table 3:** Improving hematological parameters TLC: Total leukocyte count

	Day 16	Day 20	Day 24	Day 28	Day 32
Hemoglobin (g/dL)	8.2	8.1	8.8	9.4	10.1
TLC (×10^9^/L)	3.6	3.3	4.3	5.6	8.1
Neutrophils (%)	33	34	35	46	42
Lymphocytes (%)	57	56	54	41	44
Monocytes (%)	10	10	11	13	14
Platelets (×10^9^/L)	45	46	71	102	322

The patient was discharged after 32 days and was closely followed up in the outpatient department of the hospital. Her blood counts were monitored and the outpatient course was uneventful. Informed consent from the parents and ethical board approval was obtained for this study.

## Discussion

HLH is a rare but potentially fatal non-neoplastic disease resulting from dysregulated activation and proliferation of lymphocytes, natural killer cells, and T cells. This dysregulation causes immune hyperactivation, hypercytokinemia and uncontrolled hemophagocytosis [2]. This disease is also known as hemophagocytic syndrome and is characterized by fever, hepatosplenomegaly, liver dysfunction, hyperferritinemia and hemophagocytosis throughout the reticuloendothelial system [2].

It is broadly classified as primary and secondary hemophagocytic syndrome. Primary HLH is autosomal recessive and is usually diagnosed within the first two years of life. Secondary HLH can occur at any age and it is due to the strong activation of the immune system by various malignancies, infections, autoimmune disorders, and other rheumatological diseases [2]. HLH caused by infections, also called infection-associated hemophagocytic syndrome (IAHS), makes up about 50% of all cases diagnosed [3].

Viruses are most frequently associated with secondary HLH, particularly Epstein-Barr virus (EBV) [2], but tuberculosis, malaria, leishmaniasis, and typhoid are important tropical infections that act as a trigger for IAHS [3], especially in the subcontinent. The pathophysiology of IAHS may be related to the production of high levels of activating cytokines by host lymphocytes and monocytes [3].

The diagnosis of HLH, based on the HLH-2004 diagnostic criteria, includes some common clinical, laboratory, and histochemical findings [4]. It is established if one or both of the following criteria are fulfilled:

1)      A molecular diagnosis consistent with HLH

2)      Five out of the following eight signs and symptoms:

a)      Fever

b)      Splenomegaly

c)      Cytopenia (affecting ≥2 cell lineages; hemoglobin ≤9 g/dl or ≤10 g/dl for infants <4 weeks of age, platelets 100×109/L, neutrophils <1.0×109/L)

d)     Hypertriglyceridemia (≥265 mg/dl) and/or hypofibrinogenemia (≤150 mg/dl)

e)      Hemophagocytosis in the bone marrow, spleen, or lymph nodes without evidence of malignancy

f)       Low or absent natural killer cell cytotoxicity

g)      Hyperferritinemia (≥500 ng/ml)

h)      Elevated soluble CD25 (i.e. soluble IL-2 receptor ≥2,400 U/ml)

Simple as the criteria may be, some of the clinical manifestations occur late in the disease. Since the treatment can be lifesaving, it is argued that the fulfillment of all criteria may not be necessary before initiating therapy.

Primary HLH presents early, in infancy or childhood, and can be fatal if left untreated. Secondary HLH tends to occur at an older age, in the absence of an identifiable genetic abnormality. Although the pathophysiology and presentation of both forms of HLH are similar, it is imperative to determine the type and the underlying disease [5].

Due to the worsening clinical picture and declining cell lineages along with Salmonella typhi bacteremia, we suspected HLH. With the bone marrow biopsy revealing hemophagocytosis, a diagnosis of IAHS was made and other pertinent tests were carried out to fulfill the criteria. Fever, pancytopenia, elevated ferritin, elevated triglycerides, decreased fibrinogen, and hemophagocytosis confirmed the diagnosis, as five out of the eight criteria were met.

The elevated alanine aminotransferase (ALT), aspartate aminotransferase (AST), and serum lactate dehydrogenase (LDH) levels in our patient were, although not diagnostic, suggestive of HLH, consistent with the reported incidence of over 80% in patients diagnosed with HLH [6].

Due to the rarity of the disease, no randomized controlled trials for testing potential therapies have been done. However, supportive care and treatment of the inciting infectious trigger are associated with a 60%-70% chance of recovery [7]. Corticosteroids are used to induce remission and while some may respond to the steroids, most patients require treatment with chemoimmunotherapy as well [5]. Our patient responded to antibiotics and supportive treatment alone. Previous reports have also documented success with this approach [8-10].

## Conclusions

Infections endemic to developing countries, particularly in Asia, have always been a challenge to control and eradicate. A lack of sanitation, overcrowding, and water contamination seem to compound the problem. This is a matter of concern, as many of these diseases such as typhoid fever can be complicated by secondary HLH, a rare but deadly disease with a fatal outcome. This case report intends to alert the health care practitioners about the occurrence of HLH especially when persistent fever, organomegaly, and cytopenias are encountered in the setting of an inflammatory process. HLH can overlap with other clinical conditions and malignant diseases and can obscure the features of a primary disease, making the diagnosis extremely challenging. However, timely diagnosis and the prompt initiation of therapy can drastically change the outcome and save the life of the patient.

## References

[1] Ochiai RL, Acosta CJ, Danovaro-Holliday M (2008). A study of typhoid fever in five Asian countries: disease burden and implications for controls. Bull World Health Organ.

[2] Rouphael NG, Talati NJ, Vaughan C, Cunningham K, Moreira R, Gould C (2007). Infections associated with haemophagocytic syndrome. Lancet Infect Dis.

[3] Singh Z, Rakheja D, Yadav T, Shome D (2005). Infection‐associated haemophagocytosis: the tropical spectrum. Clin Lab Haematol.

[4] Henter JI, Horne A, Aricó M (2007). HLH‐ 2004: diagnostic and therapeutic guidelines for hemophagocytic lymphohistiocytosis. Pediatr Blood Cancer.

[5] Morimoto A, Nakazawa Y, Ishii E (2016). Hemophagocytic lymphohistiocytosis: pathogenesis, diagnosis, and management. Pediatr Int.

[6] Palazzi DL, McClain KL, Kaplan SL (2003). Hemophagocytic syndrome in children: an important diagnostic consideration in fever of unknown origin. Clin Infect Dis.

[7] Fisman DN (2000). Hemophagocytic syndromes and infection. Emerg Infect Dis.

[8] Non LR, Patel R, Esmaeeli A, Despotovic V (2015). Typhoid fever complicated by hemophagocytic lymphohistiocytosis and rhabdomyolysis. Am J Trop Med Hyg.

[9] Shah PA, Rashid A, Maqbool M, Yaseen Y, Shiekh FA (2017). Enteric fever presenting as hemophagocytic lymphohistiocytosis (macrophage activation syndrome). Webmedcentral 2.

[10] Pande V, Agarkhedkar S, Tandon A, Agarwal A (2016). Haemophagocytic lymphohistiocytosis (HLH) in a case of enteric fever. Int J Bioassays.

